# The Use of Stress Cardiovascular Imaging in Pediatric Population

**DOI:** 10.3390/children10020218

**Published:** 2023-01-26

**Authors:** Sara Moscatelli, Francesco Bianco, Andrea Cimini, Mario Panebianco, Isabella Leo, Chiara Bucciarelli-Ducci, Marco Alfonso Perrone

**Affiliations:** 1 Paediatric Cardiology Department, Royal Brompton and Harefield Hospitals, Guy’s and St Thomas’ NHS Foundation Trust, London SW3 5NP, UK; 2 Cardiovascular Sciences Department, AOU “Ospedali Riuniti”, 60126 Ancona, Italy; 3 Nuclear Medicine Unit, St. Salvatore Hospital, 67100 L’Aquila, Italy; 4 Department of Cardiac Surgery, Cardiology, Heart and Lung Transplantation, Bambino Gesù Children’s Hospital IRCCS, 00165 Rome, Italy; 5 Department of Medical and Surgical Sciences, Magna Grecia University, 88100 Catanzaro, Italy; 6 Cardiac Magnetic Resonance Department, Royal Brompton and Harefield Hospitals, Guy’s and St Thomas’ NHS Foundation Trust, London SW3 5NP, UK; 7 School of Biomedical Engineering and Imaging Sciences, Faculty of Life Sciences and Medicine, King’s College University, London SW7 2BX, UK; 8 Department of Cardiology and CardioLab, University of Rome Tor Vergata, 00133 Rome, Italy

**Keywords:** stress cardiovascular imaging, pediatric cardiology, stress CMR, stress echocardiography, stress CT, nuclear imaging

## Abstract

Although not frequent in the pediatric population, ischemia could occur in children due to several congenital and acquired disease. Stress imaging is key for the non-invasive evaluation of myocardial abnormalities and perfusion defect in this clinical setting. Moreover, beyond ischemia assessment, it can provide complementary diagnostic and prognostic information in valvular heart disease and cardiomyopathies. When performed using cardiovascular magnetic resonance, it could detect, in addition, myocardial fibrosis and infarction, increasing the diagnostic yield. Several imaging modalities are currently available for the evaluation of stress myocardial perfusion. Advances in technologies have also increased the feasibility, safety and availability of these modalities in the pediatric age group. However, despite the established role of stress imaging and its increasing use in daily clinical practice, there are currently no specific guidelines, and little data are available in the literature on this topic. The aim of this review is to summarize the most recent evidence on pediatric stress imaging and its clinical application with a focus on the advantages and limitations of each imaging modality currently available.

## 1. Introduction

The evaluation of physiological or pathological changes occurring during myocardial stress is of paramount importance in the pediatric population [1]. Data collected during exercise or pharmacologically induced stress could, in fact, provide diagnostic and prognostic information in several clinical scenarios. The main indication for stress testing in adults is the evaluation of myocardial ischemia due to atherosclerotic coronary artery disease (CAD) [2,3]. Despite the fact that this disease is extremely rare in children, chest pain and myocardial ischemia evaluation still represents one of the major indications to perform stress testing in young patients, in particular in the presence of anomalous origin of coronary arteries, Kawasaki disease (KD), after cardiac transplantation or coronary artery reimplantation [1]. Beyond myocardial ischemia, stress testing can also help evaluate the hemodynamic response, pulmonary artery pressures or exercise capacity in patients with valvular heart diseases or hypertrophic cardiomyopathy. Cardiac assessment during stress conditions can also unmask myocardial dysfunction or symptoms in patients with several congenital heart diseases. In addition, the use of cardiovascular magnetic resonance (CMR) allows the detection and quantification of myocardial fibrosis and infarction as well as myocardial edema particularly useful in the acute setting. Despite all these clinical applications, there are currently no specific guidelines for the pediatric population and limited studies available in the literature. Moreover, stress imaging could be performed either with echocardiography, CMR, computed tomography (CT) and nuclear imaging, each modality having its advantages and potential drawbacks. The aim of this paper is to review current evidence on the diagnostic and prognostic role of each stress imaging modality, highlighting their strengths and weaknesses, and providing practical insights in specific congenital and acquired conditions in the pediatric age group.

## 2. Stress Echocardiography

### 2.1. Introduction

Echocardiography is a non-invasive cardiovascular imaging technique that uses ultrasound waves to generate images. This tool has the advantage of being widely accessible, radiation free and inexpensive. Echocardiography can give information regarding cardiac anatomy, systolic and diastolic function, providing a simultaneous assessment of cardiac valves. The use of a stressor (either physical or pharmacological) can complement standard assessment with information about myocardial ischemia.

In the pediatric scenario, encouraging data on stress echocardiography utility have been collected in a plethora of pathologies such as post-transplant coronary vasculopathy; anomalous origin of coronary arteries; coronary abnormalities in KD; coronary reimplantation due to arterial switch or Ross operations; valvular heart diseases; evaluation of pulmonary vascular disease; timing of intervention when a conduit dysfunction is present; evaluation of vascular obstructive disease such as coarctation; single ventricles or systemic right ventricle (RV); and previous exposure to a cardiotoxic chemotherapy agent [4].

### 2.2. Type of Stress Agents

Stress echocardiography can be performed with both a pharmacological stressor or through an exercise test. In the first scenario, two types of pharmacologic stressors are available: those that increase myocardial oxygen consumption (e.g., dobutamine) and those that cause coronary vasodilatation (e.g., dipyridamole). In general, dobutamine is used more than dipyridamole, but the choice of stressor type is linked to the experience and availability of the single center.

Dobutamine stress echocardiography (DSE) creates a positive inotropic and chronotropic effect on the heart which leads to increased myocardial oxygen demand.

Dobutamine infusion starts at 5 mcg/kg/min and is increased at 3-min intervals to 10, 20, 30, 40, and 50 mcg/kg/min. If the maximal target heart rate (HR), defined as 85% of the maximal HR for age, is not reached, a low dose of atropine (0.01 mg/kg) can be added for a maximum of 0.25 mg per dose and 1–2 mg in total. Esmolol can be used to reverse adverse reactions.

Vasodilator agents are contraindicated in advanced atrioventricular blocks, marked hypo- or hypertension, sinus bradycardia, active bronchoconstrictive or bronchospastic disease with regular use of inhalers, and known hypersensitivity to the components. Instead, contraindications to dobutamine include severe hypertension, unstable angina pectoris, severe aortic valve stenosis, complex cardiac arrhythmias, hypertrophic obstructive cardiomyopathy, myocarditis, endocarditis, or pericarditis. Finally, atropine use is not allowed in case of narrow-angle glaucoma, myasthenia gravis, and obstructive uropathy or gastrointestinal disorders. Side effects of dipyridamole are chest pain, headache, dizziness and rarely myocardial infarction, ventricular tachycardia, and transient ischemic attack. Similarly, high-dose dobutamine may cause chest pain and palpitations but also myocardial infarction, ventricular fibrillation, and sustained ventricular tachycardia [5]. Exercise stress echocardiography (ESE) can be performed with treadmill or with cycle ergometry (upright or supine); given the fact that the hemodynamic response obtained with exercise is more physiological, ESE should be preferred over DSE when feasible. The Bruce protocol is the most commonly used, consisting of three-minute stages of increasing speed and treadmill elevation [5]. In the field of stress echocardiography, a method that in recent years has been gaining enough consensus is real-time myocardial contrast echocardiography. This is widely used in the adult population, and [6] evaluates the myocardial capillary volume through the analysis of the signal intensity and the speed of the myocardial blood flow, which are altered after stress in the presence of significant coronary stenosis. This technique has demonstrated an accuracy similar to and even better to SPECT [7,8]. However, in the pediatric population, it is still not playing a leading role; new studies are needed to address this aspect.

### 2.3. Protocols

Children younger than 8 years are usually unable to cooperate and, for this reason, they are often referred to a pharmacological stress test with either general anesthesia or mild sedation; as previously mentioned, when they are older or able to cooperate, an exercise test is usually preferred.

In pharmacological stress tests, resting images are usually acquired at baseline and subsequently during stress after each increase in drug dose. In ESE, images are acquired at baseline, during incremental exercise and early within 60 sec of peak exercise, given the rapid drop in peak HR in children compared to adults.

**Figure 1:**
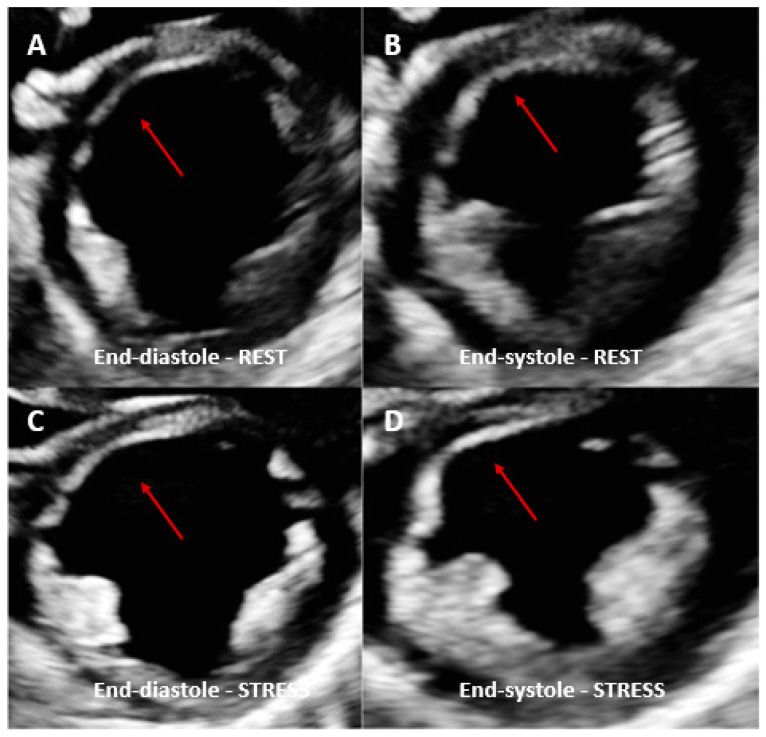
Dobutamine stress echo performed in a patient with coronary artery vasculopathy (CAV). End-diastolic rest images (**A**) demonstrated a thinned anterior septal area compared to the other myocardial segments (red arrows) and not thickening during systole (**B**). Same view acquired at peak dobutamine stress (**C**,**D**) demonstrated no significant changes in the same area with no other regional wall motion abnormalities identified. Angiography was therefore performed, showing severe left anterior descending coronary artery stenosis.

Regardless of the type of protocol used, images at rest, during stress, and in the recovery period are required. The echocardiographic images must allow the best visualization of regional and global myocardial motion (Figure 1). Generally, both the parasternal approach (long axis and short axis) and apical approach (four chambers, two chambers and long axis) should be produced. All clip sequences should be acquired with the same parameters such as depth and sector width in order to be perfectly comparable. The use of tissue harmonic imaging is preferred over a conventional imaging method due to its better image quality. Ultrasound artefacts, such as reverberation artefacts or side-lobe, should be eliminated to avoid mistakes in interpretation.

### 2.4. Limitations

Despite the several advantages named so far, echocardiography has some limitations. Suboptimal acoustic windows can significantly decrease the quality of the images acquired, particularly in patients with previous chest surgery. Furthermore, the presence of left bundle branch block, pre-existing wall motion abnormalities, malignant cardiac arrhythmias (ventricular tachycardia, complete A-V block), hemodynamically significant LV outflow tract obstruction, and severe systemic hypertension are other important contraindications.

Finally, stress echocardiography needs adequate and specialized training compared to the rest of echocardiography, limiting the access to this modality at center with higher expertise [9,10].

## 3. Stress Imaging in Pediatric Nuclear Cardiology Stress

### 3.1. Introduction

Stress testing in nuclear cardiology for myocardial perfusion imaging involves a stress test (to assess myocardial perfusion) and a scintigraphic scan of the heart, with the administration of a radiopharmaceutical. Scintigraphic images are obtained after myocardial stress and in rest conditions [11] with the myocardial uptake of the radiolabeled tracer that depends on coronary flow, cell membrane integrity, and cell viability [11].

**Figure 2:**
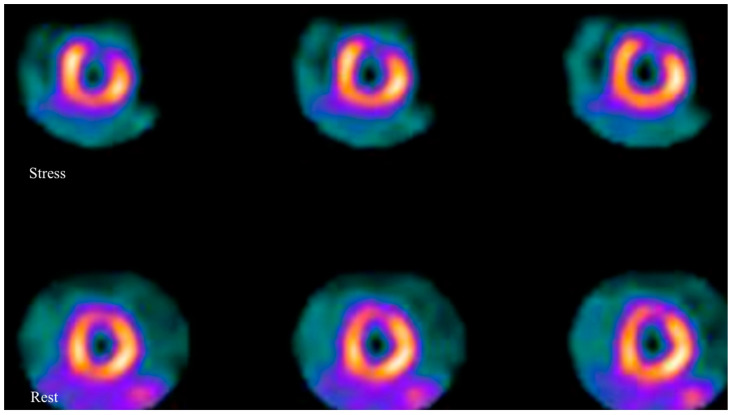
Axial images of a myocardial scintigraphy performed in a pediatric patient with KD: images show a severe reversible perfusion defect in the anterior wall of left ventricle, which is suggestive for myocardial ischemia.

The main clinical indications of stress testing imaging in pediatric nuclear cardiology are represented by KD to evaluate myocardial ischemia and/or myocardial infarction (Figure 2) [12,13], the assessment of coronary perfusion after the arterial switch operation for transposition of great arteries [14], the post-operative follow-up of children with anomalous origin of the left coronary artery from the pulmonary artery (to assess the extension of ischemic myocardium) [13], the evaluation of residual ischemia after surgery in pediatric patients with tetralogy of Fallot [13], the assessment of myocardial function in pediatric patients with cardiomyopathies [15], and the evaluation of children with chest pain when electrocardiograms and/or echocardiography detect abnormalities [16].

### 3.2. Type of Stress Agents

In nuclear cardiology, stress testing may be performed with physical stressors (such as cycle ergometer) or pharmacological stressor or the combination of the two. Pharmacological stress is usually preferred in children since tests with physical stressors require high compliance from patients [13].

Adenosine is the most used pharmacological stressor in pediatric patients [9]: adenosine is a nucleotide, administered intravenously (with continuous infusion, 140 mcg/kg/min for 6 min), that causes coronary vasodilatation binding A2a receptors [17]. In clinical practice, adenosine is used for its short half-life in the bloodstream (10 s): hence, side effects such as bronchospasm (due to activation of A2B and A3 receptors), atrioventricular (AV) block (due to activation of A1 receptors) and peripheral vasodilatation (due to activation of A2B receptors) are mild and quickly disappeared [18,19]. Other minor side effects are represented by flushing, dyspnea, and nausea [19]. Xanthine (contained in chocolate) interferes with adenosine; hence, products containing xanthine should be avoided for at least 12 h prior to the exam [19]. Dipyridamole is another pharmacological stressor used for stress testing imaging in pediatric nuclear cardiology [20,21]: this is a coronary vasodilator that acts by inhibiting intracellular and deamination of adenosine [22]. Side effects are the same as described for adenosine, and products with xanthine should be avoided prior to the test. Compared to adenosine, dipyridamole has a longer half-life (30–45 min) [22]. Regadenoson, a high selective A2a receptor agonist (with a weaker affinity for A1 receptors), represents a valid alternative to adenosine and dipyridamole. Whilst widely used in adults, to the best of our knowledge, there are no reports on its utilization in pediatric nuclear cardiology, but the feasibility of regadenoson as a pharmacological stressor in stress imaging is described in studies with myocardial stress perfusion magnetic resonance [23,24].

### 3.3. Protocol

Stress testing imaging may be obtained by means of single-photon emission computed tomography (SPECT) or positron emission tomography (PET) after the injection of radiopharmaceuticals: for the injected dose, it is important to underline that the ALARA (“as low as reasonably achievable”) principle must be followed in order to minimize radiation exposure. In general, a dose reduction could be performed with algorithms (for example “European association of nuclear medicine (EANM) dose calculator”) or with the EANM Dosage Card [13,25,26].

SPECT images are acquired with a gamma camera: a two-head gamma camera—preferably one with an L configuration, using a 180-degree rotation from the right anterior oblique to left posterior oblique; moreover, the utilization of pinhole or converging collimators may be useful in pediatric patients in order to optimize SPECT imaging with an improved spatial resolution [27].

The most used SPECT radiopharmaceuticals in stress testing imaging are ^99m^Tc-2-methoxyisobutylisonitrile (^99m^Tc-Sestamibi), ^99m^Tc-1,2-bis[bis(2-ethoxyethyl) phosphino] ethane (^99m^Tc-Tetrofosmin), and thallium-201 (^201^Tl) chloride. ^99m^Tc-Sestamibi and ^99m^Tc-Tetrofosmin are cationic complexes that diffuse passively through the capillary and cell membrane: the myocardial uptake of these radiopharmaceuticals augments with increase in perfusion, reflecting myocardium viability [27]. ^201^Tl is a analogue of potassium: its uptake in myocytes is mediated by sodium/potassium adenosine triphosphate (ATP) transporter [24]. In pediatric patients, ^99m^ Tc-Sestamibi and ^99m^Tc-Tetrofosmin have better physical characteristics in comparison to 201Tl chloride: the main drawbacks of ^201^Tl are a higher physical half-life (the 201Tl half-life is about 73 h, while the 99mTc half-life is about 6 h) and a higher radiation burden for children. Moreover, 99mTc presents a better gamma energy emission (140 keV), which is seen as more appropriate when evaluating small hearts of pediatric patients [13,28].

As mentioned before, scintigraphic images are obtained after myocardial stress and in rest conditions: SPECT with ^99m^Tc labelled radio compounds needing two injections of ^99m^Tc- Sestamibi or ^99m^Tc-Tetrofosmin, one for both phases. On the other hand, SPECT with ^201^Tl chloride requires only one injection of the radiopharmaceutical due to its redistribution [28].

PET imaging has a higher spatial resolution in comparison to SPECT; furthermore, this imaging technique may provide an accurate quantitative data of myocardial blood flow. Nonetheless, few reports in the literature concerning stress testing imaging in pediatric nuclear cardiology are available: these reports are mainly focused on PET with [^13^N] Ammonia ([^13^N]NH3) in children after arterial switch operation for the transposition of great arteries [29] and in pediatric patients with coronary abnormalities [30]. An onsite cyclotron for the synthesis of [^13^N]NH3 is required due to the short half-life of this radiopharmaceutical (9.96 min) [31]: this is one of the most important limitations for the utilization of PET with [^13^N]NH3 in clinical practice.

### 3.4. Limitations

As regards the injected dose in children, the minimization of radiation exposure is mandatory: the ALARA (“as low as reasonably achievable”) principles should be followed, and dose reduction may be performed with algorithms [13,24].

The detection of lesions in small hearts may be challenging in pediatric nuclear cardiology: as suggested before, the utilization of converging or pinhole collimators in the acquisition process may be useful (especially in infants) to improve spatial resolution. Moreover, nuclear medicine physicians should be aware of the possible overestimation of parameters deriving from Quantitative Gated SPECT (QGS), such as left ventricular ejection fractions, often higher in small hearts [25,32].

## 4. Stress Cardiovascular Magnetic Resonance

### 4.1. Introduction

CMR is an advanced cardiovascular imaging method that not only allows the assessment of cardiac anatomy, function, hemodynamics, and tissue characteristics but also, through the use of stress techniques, the assessment of myocardial viability and perfusion [33,34]. In addition, CMR acquires all data non-invasively and without the use of ionization radiation. In recent years, many scientific studies in the adult population have established the safety and accuracy of stress perfusion CMR in identifying myocardial ischemia [35,36,37,38]. International guidelines on the use of cardiovascular magnetic resonance in pediatric population also describe the use of stress perfusion in this subset of patients [39,40].

### 4.2. Stress CMR: Adult vs. Pediatric Populations

The main studies that have confirmed the clinical role of stress CMR to assess myocardial ischemia in the adult population are MR-IMPACT I and II both multicentric studies showing the superior sensitivity and inferior specificity of stress CMR compared to SPECT for CAD [35,36]; these were followed by randomized clinical trials CE-MARC I and CE-MARC II which documented the increased diagnostic accuracy of stress CMR versus SPECT [37,38]. Finally the MR-INFORM randomized trial showed that stress CMR is non-inferior to fractional flow reserve (FFR) in adult patients with stable angina [41]. This evidence contributed to the Class I recommendation for stress CMR in patients with chest pain and intermediate risk of CAD both in the 2019 European Society Guidelines for the diagnosis and management of chronic coronary syndrome and in the 2021 American Guidelines for the evaluation of chest pain [3,42].

Indeed, it is difficult to extrapolate this CMR experience from adult CAD to the younger population given also the different pathological processes subtended to ischemia in the pediatric population. On the other hand, the different abilities of the coronary vasculature and the myocardium in the pediatric population to adapt to ischemia, such as the development of collateral blood supply, can masquerade an underlying pathology [43,44,45,46]. However, encouraging data on the feasibility and accuracy of stress CMR in coronary artery diseases have been shown in children, but for ages below one year, the diagnostic image quality is still limited [47,48,49]. Encouraging results have also been shown for assessing the increase in the right ventricle stroke volume in patients with TGA post ASO or congenitally corrected TGA or patients with Tetralogy of Fallot before repair [46,47,48,49,50,51,52].

### 4.3. Type of Stress Agents

Stress CMR can be performed either through physical or pharmacological stressors. In the first case, patients cycle while laying inside the CMR scanner to reach 85% of the maximum theoretical heart rate (HR). In the second scenario, a vasodilator stress perfusion can be performed using adenosine, regadenoson, dipyridamole, and adenosine triphosphate (ATP), aiming at obtaining an increase in HR by 10 bpm and or a systolic blood pressure drop >10 mmHg [33]. The 85% of the maximum theoretical HR criterion can be used instead when evaluating the response to an inotropic agent [33]. Contraindication and collateral effects have been extensively addressed in the previous sections.

### 4.4. Protocol

**Figure 3:**
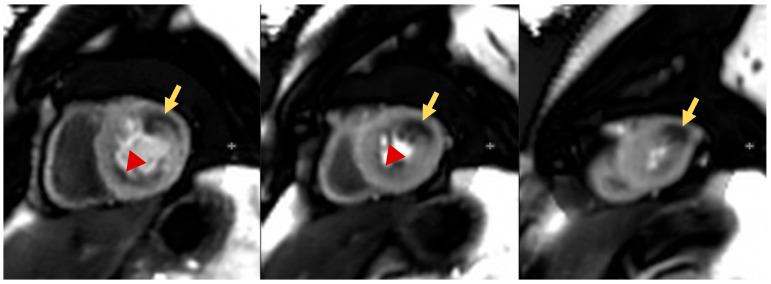
Pediatric patients affected by KD with multiple aneurysms. The stress perfusion sequences show significant myocardial perfusion defects in the basal to mid anterolateral and apical lateral walls (likely circumflex territory, yellow arrows). Separately, there is also a mild subendocardial perfusion defect in the basal to mid inferior wall and inferoseptum (likely right coronary artery territory, red arrow heads).

Stress CMR can be performed either by inducing vasodilatation (vasodilator stress) or increasing heart rate (HR) aiming at reaching 85% of the maximal theoretical HR. Once hyperemia or HR threshold is achieved, a gadolinium-based contrast agent (GBCA) is injected into a peripheral vein. GBCA transit is visualized through a series of T1-weighted CMR images acquired during every heartbeat. Given the characteristic of shortening the T1, GBCA is visualized passing through heart chambers and then through the myocardium with a signal proportional to its concentration. The stress perfusion images are acquired in the short axis at basal, mid and apical levels of the left ventricle. Once the stress sequences are performed, the infusion of the stressor is interrupted, in the case of a vasodilator, an antidote such as aminophylline could be theoretically administered reverting the vasodilatation effect, but this is seldomly used. Rest perfusion images can be performed 10–15 min after the stress images using the same image positions (slices) so that the stress/rest myocardial segments are comparable. The perfusion defect is visualized as a reduced signal (reduced contrast perfusion) in the subendocardium which can persist for more than 3–4 heartbeats after the enhancement of the left ventricular cavity and for several seconds after the contrast washes out from the left ventricle (Figure 3). Fifteen to twenty minutes after contrast injection, late gadolinium enhancement (LGE) sequences are acquired to detect the possible presence of myocardial fibrosis/scarring. These data are important for interpreting perfusion results because perfusion defects can be either due to myocardial ischemia or to a pre-existent myocardial infarction that will be present in LGE sequences. Sometimes, artefacts can present in the form of a circumferential dark rim in the sub-endocardium layer simulating a genuine inducible myocardial; thus, expertise in acquiring and interpreting these images is required. Dark rim artefacts are typically persistent during both resting and stress conditions, while real inducible perfusion defects appear only during stress [53].

### 4.5. Limitations

Despite the great advantages of CMR, this modality is not without limitations. First, it is a long exam that requires a great level of compliance which can be a challenge in the pediatric population. However, the protocol can be tailored and reduce image acquisition only to the essential images, thus significantly reducing the length of the examination. Indeed, a recent study has shown in the adult population how the workflow improvement decreases the duration of the CMR examinations and allows wider access to clinical routine [54].

The need of general anesthesia or sedation in younger patients also represents a possible limitation [55]. Using general anesthetic can change the physiological body status, potentially making it more difficult to achieve a good level of stress giving false negatives. In addition, although rare, there is the potential risk of life-threatening adversity, requiring therefore adequately trained staff. Furthermore, not all devices such as pacemakers or ICDs are MRI conditional, and this can generate concerns for clinicians when performing MRI [56,57,58]. However, there are recent data in the adult population that CMR is safe both in MR conditional and MR non-conditional (legacy) devices [59,60]. In addition, the use of GBCA is not without any risks; in a very small percentage of patients, it has been associated with allergic reactions (mainly mild). The use of GBCA in severe kidney dysfunction (eGFR < 30 mL/min/1.73 m^2^) has been also associated with nephrogenic systemic fibrosis (NSF). Lastly, there is some evidence in patients undergoing non-cardiac MRI documenting that multiple administration of GBCA in short time frames has been linked to the accumulation of gadolinium in the basal ganglia of the brain [61].

## 5. Stress Computed Tomography Perfusion Imaging

### 5.1. Introduction

Computed tomography (CT) coronary angiography (CTCA) is a valuable, non-invasive, three-dimensional modality for coronary imaging that could help detect the coronary artery (CA) stenosis or calcifications in adults and anomalies or abnormalities in children and neonates [62,63].

### 5.2. Protocol

In this setting, ECG-gated acquisitions are essential, both prospective and retrospective. The synchronization is obtained utilizing the ECG R-R interval, expressed in milliseconds (ms), that represents the percent of the R-R interval after or before the R wave. [64]

Retrospective gating is achieved utilizing conventional spiral imaging, with volume data acquisition, and it is utilized when information about the entire cardiac cycle and volume are needed. The scan is over several heart beats (usually 6-to-10) with the simultaneous recording of the ECG at a very low pitch to allow for image reconstruction at any phase of the cardiac cycle. Subsequently, the cardiac phase less affected from cardiac motion artifacts can be chosen for the image interpretation. In this way, it is possible to image the full-length coronary arteries anatomy and systolic/diastolic variations can be studied [62,64].

On the contrary, if only diastolic anatomic details are needed to be studied, a prospective CTCA imaging is recommended. Prospective triggering is commonly performed in combination with axial (step and shoot) scanning module, with data acquisition triggered by a pre-specified time, corresponding to a certain phase of the cardiac cycle (i.e., the diastole), and accomplished over several heart beats until the whole heart is imaged. Second-generation 128-slice dual-source CT systems permit an acquisition time of 250 ms, approximately. Therefore, CT can be performed in a small portion of a single heartbeat. The high-pitch scan (3.2–3.4 of pitch) can be limited only to the diastole, with a very low patients radiation dose exposure. The latter is extremely important when performing prospective ECG-gated CTCA both in children and neonates [63,64].

**Figure 4:**
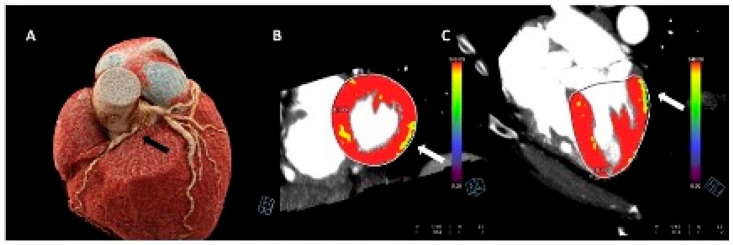
Stress-computed tomography perfusion imaging of a 15-year-old boy with an anomalous origin of the right coronary artery from the left sinus of valsalva. The black arrow indicates the coronary artery abnormality (Panel (**A**)). The images with arrows (**B**,**C**) indicate stress-induced perfusion defects of the infero-lateral wall of the left ventricle.

The combination of CTCA with myocardial perfusion imaging offers a unique and comprehensive tool for the assessment of CA anatomy and physiology. Myocardial perfusion on CTCA is based on the distribution of iodinated contrast agent during its first pass through the myocardium. Because contrast agent distribution is determined by the arterial blood supply, myocardial perfusion defects can be identified as hypo-attenuated areas containing low amounts of contrast (Figure 4) [65].

In neonates and young children, non-ionic iodinated contrast agents are injected at a dose of 2 mL/kg to a maximum amount of 100 mL. The injection could be at 1 mL/s but might be increased to 2 mL/s in patients with a large intracardiac communication (i.e., large ventricular/atrial septal defects). In adults, 1.5 mL/kg is generally injected at a rate of 3–4 mL/s. In ECG-synchronized CT, the scan delay is determined through the bolus-tracking technique. The region of interest (ROI) in pediatric patients is positioned in the left ventricle at a threshold attenuation of 200 HU. In adult patients, the ROI is sited in the ascending or descending aorta, and the attenuation threshold is set at 140–180 HU [64].

Dual-source CTCA scanners implementation (3.2–3.4 of pitch, 66–75 ms of temporal resolution, and 0.4 × 0.6 mm of spatial resolution) drastically diminished cardiac and respiratory motion artifacts. They offer diagnostic images even at higher heart rates (>80 bpm). Therefore, exams performed for most indications can be acquired without sedation and during free breathing [63,64].

As with other myocardial perfusion imaging exams (i.e., SPECT or PET), the myocardial CT perfusion protocol consists of an acquisition during stress, which is caused by the administration of pharmacological agents such as adenosine, to induce vasodilation. A resting acquisition, and a third delayed acquisition, which can be performed in cases where a late-contrast-enhanced evaluation of myocardial scars is desired. Heart rhythm and symptoms are monitored throughout the whole exam [65,66].

In detail, after scouting images and testing bolus, adenosine can be administrated at an infusion rate of 140 μg/kg/min [67]. Three minutes (3 min) later, a contrast-enhanced stress perfusion scan can be acquired using retrospective gating. Once the heart rate normalized, or returns to the baseline, a rest perfusion scan can be acquired. This can be a prospective contrast-enhanced scan [67]. Around 7 min later, a low tube voltage, prospectively triggered delayed enhancement scan can be acquired in case of a late-contrast-enhanced evaluation for myocardial scars is needed. Then, images for CA anatomy, perfusion and scar can be evaluated and postprocessed with a color scale. Nominally, a perfusion defect under pharmacological stress that reverses at rest is a stress-induced ischemia. Conversely, an irreversible defect is characteristic of myocardial infarction [65,66,68].

### 5.3. Limitations

Despite the premises and the promising techniques implemented in adults, several issues should be addressed and discussed when dealing with pediatric patients, especially when neonates. Firstly, stress imaging techniques cannot be applied/implemented when sedation is contextually administrated. Therefore, a certain collaboration during myocardial perfusion CTCA imaging is needed; this depends on the patients’ age and compliance; children are usually more compliant when >6–7 years old. If they are >4 years old, each exam should be tailored to every single patient. In addition, given the incremental radiation dose exposure for stress CTCA imaging, the risks and benefits of each exam should be carefully balanced. Finally, the stress SPECT or PET protocols with 6-min adenosine infusion have been demonstrated to be superior to 3-min ones in the detection of coronary artery disease (CAD) in adults, while uncertainty persists regarding the relationship between ischemia and exercise-induced abnormalities in myocardial perfusion imaging in children. Ischemic echocardiographic and ECG changes may have a different stage in pediatric patients with Kawasaki disease, coronary arteries abnormalities (both origin and course) or myocardial bridges and fistulas. To date, it is unclear whether the “ischemic cascade” concept that has been found to be so helpful in the diagnosis and management of typical adult atherosclerotic CAD applies with equal validity to these anatomically and physiologically distinct conditions [68]. Therefore, more and more studies are warranted in helping to define the role of stress CTCA in these settings.

All advantages and disadvantages of the single cardiovascular imaging method are summarized in Table 1.

## 6. Clinical Applications of Stress Imaging in Children

### 6.1. Arterial Switch Operation (ASO)

Transposition of the great arteries (TGA) is a complex congenital heart disease characterized by ventricular arterial discordance that is treated in the first weeks of life with an ASO operation where the aorta is transacted and reconnected to the left ventricle (LV) and pulmonary artery to the RV. In this delicate procedure, coronary buttons are created and reimplanted onto the aorta [69]. This last maneuver has been associated in the long-term follow-up with coronary occlusion leading to death. Indeed, late intravascular ultrasound assessment has revealed proximal eccentric intimal thickening in coronary arteries, suggesting the development of early atherosclerosis in reimplanted coronary arteries [70].

Assessment with stress imaging is therefore mandatory to evaluate ischemia in this clinical scenario. Hui et al. demonstrated in a cohort of 31 ASO patients that DSE was able to unmask wall motion abnormalities in 74% of patients (n = 23). Myocardial perfusion scan was subsequently performed in 22 patients, confirming perfusion defects in the dysfunctional segments in 77% (n = 17) of them [71]. Another study performed on 36 patients demonstrated safety and feasibility of regadenoson stress CMR in children and young-adults with previous ASO, showing excellent agreement with cardiac catheterization [23,72].

Although there is no consensus regarding the appropriate timing for these examinations, Sterrett et al. recommended serial myocardial perfusion stress testing during periods of growth; every 3 years and before high school sports participation [73].

### 6.2. Heart Transplant

Coronary artery vasculopathy (CAV) in one of the most common consequences of heart transplantation, with angiography currently representing the gold standard for the assessment of this disease. However, this method is invasive with possible, even life-threatening, complications. Stress imaging therefore represents a valuable alternative in this setting and is currently recommended by expert consensus [74]. DSE has been safely use to detect CAD with a sensitivity reported between 70 and 80% and a specificity up to 88% if IVUS is used as a gold standard in adults [5,74,75]. In children, these data seem to be confirmed although with higher variability between studies [76,77]. Exercise protocols have demonstrated a very poor sensitivity in this setting in adults due to the denervation and consequent limited heart rate increase during exercise. A single center study conducted on 47 patients who were pediatric cardiac transplant recipients showed instead higher ESE sensitivity (89%) and specificity (92%) [78]. Either DSE or ESE are recommended to help extend the interval between angiograms in the asymptomatic pediatric transplant recipient [6,79]. CTCA is a non-invasive alternative to angiography to assess coronary anatomy with good accuracy [80]. Finally, regadenoson stress CMR was safely performed in 26 heart transplant pediatric patients with lower values of myocardial perfusion reserve index when compared to healthy controls [80].

### 6.3. Kawasaki Disease

KD is a systemic inflammatory vasculitis that tends to affect small vessels and that frequently involves coronary arteries causing coronary artery dilation and aneurysms [81]. It is considered the first cause of myocardial ischemia in the pediatric population. DSE has demonstrated excellent specificity and negative predictive value in this setting. Zilberman et al. found that DSE was useful to distinguish high-risk patients from lower-risk patients [82]. DSE also provides independent prognostic information with cumulative event-free survival rate at 15 years of follow up of 25% of young patients with Wall Motion Score Index > 1.25 and 91.7% in patients with Wall Motion Score Index <1.25 [83]. In addition, Pahl et al. have shown a good correlation between ESE findings and KD and coronary abnormalities detected at angiography [84]. Assessment of coronary artery aneurysm is feasible with CMR angiography [85]. In addition, stress CMR is feasible to assess ischemia due to KD and demonstrated lower values of myocardial perfusion reserve index compared to healthy children [24,45,86,87].

### 6.4. Others Clinical Indications

Anomalous origin of a Coronary Artery (AOCA) is one of the most common causes of sudden cardiac death, with intramural or inter-arterial course of the left coronary artery being the highest risk variants [88]. CCTA is key for non-invasive assessment of the coronary artery anatomy, but stress imaging could help in risk stratification and surgical planning. Both ESE and stress CMR are used routinely for this purpose, with a demonstrated overall agreement of 79% between CMR and Fractional Flow Reserve [89]. Stress imaging can be also useful to assess dynamic gradient in coartation, re-coartation and hypertrophic cardiomyopathy [90,91]. Of note, vasodilator stress has limited value to assess ischemia in AOCA or myocardial bridges. In fact, due to the physiopathological mechanism of ischemia in these patient populations (compression of coronary vessels), a positive chronotropic and inotropic stressor (exercise or dobutamine) is required to elicit ischemia.

Although atherosclerotic disease is very rare in children, patients with homozygous familial hypercholesterolemia can have severe coronary artery disease with myocardial infarction even at a pediatric age [92]. For these reasons, serial stress imaging assessment could be helpful in evaluating these high-risk patients [84]. In addition, despite rest echocardiography being the method of choice to assess the etiology and severity of the valvular disease, stress CMR can provide additional information, guiding the timing of surgery [93]. However, despite wide potential application, there are currently very little data regarding the use of stress imaging to stage valvular heart disease in the pediatric patients.

Coronary microvascular dysfunction (CMD) may represent one of the causes of chest pain even in pediatric patients, particularly in the context of congenital heart disease or long-standing type 1 diabetes mellitus [94]. Although the gold standard for CMD diagnosis still remains coronary flow reserve (CFR) and index of microcirculatory resistance (IMR) invasive measurements during coronary angiography [95], several non-invasive imaging alternatives have been so far provided. Cardiac PET is the most validated imaging modality for the non-invasive assessment of CMD, allowing the quantification of myocardial blood flow (MBF) and myocardial perfusion reserve (MPR). The same parameters could be measured by quantitative perfusion stress CMR using a recently developed respiratory motion-corrected myocardial perfusion method that demonstrated good correlation against PET and invasive measurements [95,96]. CFR can be also assessed using transthoracic doppler echocardiography as a ratio between coronary blood flow velocity in the left anterior descending artery during hyperemia and at rest. Less used is the ultrasound-based myocardial contrast enhancement technique that exploits intravenous contrast microbubbles to estimate myocardial blood flow [95]. Assessing CMD by CT is technically feasible but requires exposure to radiation and is currently limited to few highly specialized centers. However, despite all the evidence provided so far about the usefulness of non-invasive imaging evaluation of CMD, there is currently limited data regarding their use in the pediatric population, which may warrant further studies to establish their diagnostic and prognostic role [97].

Of note, in the adult population, the measurement of FFR retrospectively or prospectively from simple invasive coronary angiography images has led to the defniton of the quantitative flow ratio (QFR). This is a new important parameter to detect the presence of inducible ischemia. However, in the pediatric population where the main coronary disease is not coronary arteriosclerosis, this has still not been applied. Given its great results, it could be an important future resource that could be used in these patients [98,99].

## 7. Conclusions

Evaluation of cardiac performance and perfusion during stress condition is key in several congenital and acquired conditions. Although the role of stress testing is already established in the pediatric population, there are currently no specific clinical guidelines for this age group. Different imaging modalities can be used for stress evaluation, each one with its advantages and disadvantages. Echocardiography is a widely available, radiation-free, and low-cost technique that can be used during either pharmacological or exercise stress. Despite representing the first-line stress imaging modality, its accuracy could be significantly reduced in patients with poor acoustic windows, particularly after chest surgery. Nuclear imaging can overcome this drawback; technological evolution with improved detectors and the advent of hybrid machines (SPECT/CT and PET/CT) have reduced the amount of radiation exposure with improved diagnostic accuracy. Similarly, the advent of dual source CT scanners along with high-pitch modality have significantly reduced the overall amount of radiation needed to obtain CTCA and stress perfusion CT in children. However, acquiring stress images with CT could be particularly challenging in non-compliant children, and the modality requires the use of an iodinated contrast agent which is contraindicated in patients with severe chronic kidney disease. Stress CMR represents a valuable non-invasive imaging alternative with very few limitations represented by the high cost of the modality, long acquisition time and the relative contraindication in patients with some metallic devices. Its unique tissue characterization properties along with the complete absence of radiation make CMR particularly appealing when assessing young patients, often requiring serial imaging evaluation. The options available are therefore several and always more often integrated in a multi-modality approach. Clinicians should be aware of the strengths and limitations of each modality in order to choose the best technique and one which is most likely able to answer the clinical question. Finally, beyond diagnostic performance and limitations, particularly in the pediatric age group, local expertise and availability are crucial factors when deciding which is the most suitable stress test to perform.

## Figures and Tables

**Table 1:** Advantages and disadvantages of each cardiovascular imaging method. * Unless performed in expert centers where non-MRI conditional devices are performed in adults.

Modality	Advantages	Disadvantages
Echocardiography	Low cost, widely available, radiation free Provides simultaneous assessment of dynamic gradients and valvular heart disease	Limited image quality in poor acoustic windows High operator dependence
Nuclear Imaging	Good spatial resolution Can assess cardiac metabolism Can complement other modalities in hybrid machines	Requires radiation exposure Requires management of radioactive tracers
Computed Tomography (CT)	High spatial resolution Provides simultaneous assessment of coronary anatomy Dual source CT scans significantly reduced radiation exposure and improved image quality even at higher heart rates The 16-cm wide-detector CT permits single-heartbeat and free-breathing scans with low radiation doses and good diagnostic image quality	Requires radiation exposure and the use of iodinated contrast agent Requires cooperation and is therefore limited to more compliant children Single heart-beat scans are limited in stress imaging
Cardiovascular Magnetic Resonance (CMR)	Gold standard for volumetric assessment Radiation free Provides complimentary tissue characterization	Contraindicated in patients with non-conditional devices * Longer acquisition time and cooperation (breath-holding) required

## Abbreviations

ALARA	as low as reasonable achievable
ASO	arterial switch operation
ATP	adenosine triphosphate
AV	atrioventricular
CA	coronary artery
CAD	coronary artery disease
CAV	coronary artery vasculopathy
CFR	coronary flow reserve
CMD	coronary microvascular dysfunction
CMR	cardiovascular magnetic resonance
CT	computed tomography
CTCA	computed tomography coronary angiography
DSE	dobutamine stress echocardiography
EANM	European Association of Nuclear Medicine
ESE	exercise stress echocardiography
FFR	fractional flow reserve
GBCA	gadolinium-based contrast agent
HR	heart rate
ICDs	implantable cardioverter defibrillators
IMR	index of microcirculatory resistance
KD	Kawasaki disease
LGE	late gadolinium enhancement
LV	left ventricle
MBF	myocardial blood flow
MPR	myocardial perfusion reserve
MRI	magnetic resonance imaging
Ms	milliseconds
NSF	nephrogenic systemic fibrosis
PET	positron emission tomography
QGS	quantitative gated SPECT
RCA	right coronary artery
ROI	region of interest
RV	right ventricle
SPECT	single-photon emission computerized tomography
TGA	transposition of the great arteries
